# Association between Vitamin D Status and Mortality among Adults with Diabetic Kidney Disease

**DOI:** 10.1155/2022/9632355

**Published:** 2022-05-09

**Authors:** Feng Xu, Hongyu Lu, Tianwen Lai, Ling Lin, Yongsong Chen

**Affiliations:** ^1^Department of Respiratory and Critical Care Medicine, The First Affiliated Hospital of Shantou University Medical College, Shantou, Guangdong 515041, China; ^2^Clinical Research Center, The First Affiliated Hospital of Shantou University Medical College, Shantou, Guangdong 515041, China; ^3^The Affiliated Hospital of Guangdong Medical University, Zhanjiang, Guangdong 515041, China; ^4^Department of Rheumatology, The First Affiliated Hospital of Shantou University Medical College, Shantou, Guangdong 515041, China; ^5^Department of Rheumatology, Shantou University Medical College, Shantou 515041, China; ^6^Department of Endocrinology, The First Affiliated Hospital of Shantou University Medical College, Shantou, Guangdong 515041, China

## Abstract

**Objective:**

Emerging evidence demonstrates that vitamin D status contributes to the incidence of diabetic kidney disease (DKD). However, the causal relationships between vitamin D and mortality among individuals with DKD are inconclusive. Our study is aimed at exploring the relationship between serum 25-hydroxyvitamin D (25(OH)D) concentrations and mortality among adults with DKD. *Research Design and Methods*. Our study included 1,202 adult participants with DKD from the National Health and Nutrition Examination Survey (NHANES) 2001–2014. Cox and competing-risks regression were used to estimate hazard ratios (HRs) and 95% CIs for associations between 25(OH)D concentrations and survival.

**Results:**

The overall mean serum 25(OH)D concentration was 55.9 ± 26.3. Vitamin D deficiency (25(OH)D < 50 nmol/l), insufficiency group (50 ≤ 25(OH)D < 75 nmol/l), and sufficiency group (25(OH)D ≥ 75 nmol/l) were observed in 552 (45.9%), 409 (34.0%), and 241 (20.0%) participants, respectively. Higher levels of vitamin D were significantly associated with improved all-cause and nonaccident- and malignant neoplasm-cause mortality among individuals with DKD after adjusting for the potential confounding factors.

**Conclusions:**

We observed widespread vitamin D deficiency or insufficiency in DKD patients. Higher 25(OH)D values were significantly correlated with lower risk of mortality after adjusting for confounding variables.

## 1. Introduction

Diabetic kidney disease (DKD) is the leading diabetic complication, characterized by proteinuria, glomerulosclerosis, and progressive renal insufficiency [1, 2]. Approximately one third of type II diabetic patients develop DKD and represent the most frequent cause of end-stage renal disease worldwide; moreover, the incidence of DKD continues to rise [3, 4]. The presence of DKD has an extremely adverse impact on quality of life and risk of mortality and becomes a major global medical, societal, and economic burden [5, 6].

Vitamin D plays a pivotal role in the maintenance of calcium and phosphate metabolism in bone health [7]. Besides that, recent evidence focuses on the possible effects of vitamin D deficiency for a wide range of diseases and conditions such as cardiovascular disease, tuberculosis, metabolic syndrome, diabetes, and certain types of cancer [8–10].

Although emerging evidence from some epidemiological studies described that lower vitamin D deficiency was linked to elevated risk of DKD [11–13], the evidence on the relationship between vitamin D status and risk of mortality among DKD was limited and somewhat mixed. Vitamin D and its analogue supplementation were shown to reduce renal cell injury and demonstrated a significant survival benefit for DKD patients [14]. Vitamin D deficiency or insufficiency is common worldwide in all age subgroups [15]. Serum vitamin D (25-hydroxyvitamin D, 25(OH)D) concentration as a biomarker for vitamin D status has been widely used. Therefore, the objective of our current study, using a nationally representative data of US participants with DKD from National Health and Nutrition Examination Surveys (NHANES), was to examine the potential association between 25(OH)D and mortality.

## 2. Research Design and Methods

### 2.1. Study Population

The NHANES provided cross-sectional data to assess nutritional and health status among the US noninstitutionalized civilian population. Our study utilized data from the continuous NHANES survey from seven survey cycles, conducted between 2001 and 2014 with a total of 72,126 participants. Participants (aged ≥20 years) with 38,913 eligible participants were included in the study population. Diabetes was a complex disease defined by the following criteria: self-reported doctor diagnosis of diabetes or glycated hemoglobin A_1c_(HbA_1c_) ≥ 6.5% due to the changes in the diagnostic criteria over time. DKD diagnosis was accessed by measurement of diabetes with impaired estimated glomerular filtration rate (eGFR < 60 ml/min/1.73 m^2^), albuminuria (albumin-to-creatinine ratio ≥ 30 mg/g), or both. Participants that are pregnant women, cancer patients, and participants who were missing a vitamin D measurement were excluded, and 1,202 participants were finally included in the study population.

### 2.2. Assessment of Serum 25(OH)D

In this analysis, the concentrations of serum 25(OH)D from participants in NHANES 2001–2006 were measured by the DiaSorin radioimmunoassay kit (Stillwater, MN). Serum samples from participants in NHANES 2007-2014 were analyzed by the CDC laboratory for 25(OH)D metabolites by liquid chromatography–tandem mass spectrometry (LC-MS/MS) or expressed as LC-MS/MS equivalent.

### 2.3. Study Variables

To identify vitamin D status as an independent risk factor for all-cause mortality, the following potential confounders were also extracted and adjusted from the NHANES database. Continuous variables included age, BMI, family income-poverty ratio, glucose, glycohemoglobin, triglyceride, LDL cholesterol, HDL cholesterol, total cholesterol, blood urea nitrogen, and creatinine; categorical variables included sex, race, and educational level.

### 2.4. Assessment of Mortality

All-cause mortality data was determined by linkage to the National Death Index through 25 February 2019.

### 2.5. Statistical Analysis

Participants were categorized into quartiles based on the values of 25(OH)D (<25.0, 25.0–49.9, 50.0–74.9, and ≥75 nmol/l) according to the Endocrine Society Clinical Practice Guidelines [16, 17]. The adjusted hazard ratios (HRs) and 95% confidence intervals (95% CIs) were calculated and presented using Cox proportional hazards regression model. Accidents (unintentional injuries) and malignant neoplasms were considered competing events. This observational study used three models in compliance with the Strengthening the Reporting of Observational studies in Epidemiology (STROBE) guidelines [18]. An initial model adjusted for age, sex, and race (model 1). Model 2 was further adjusted for variables used in model 1 plus other risk factors for BMI, education levels, and family income-poverty ratio. Model 3 was adjusted for all variables in model 2 plus other risk factors for glucose, glycohemoglobin, total cholesterol, HDL, LDL, triglyceride, creatinine, and blood urea nitrogen. We generated Kaplan–Meier survival curves for all-cause mortality stratified by vitamin D status. Smooth curve fitting (penalized spline method) was used to examine the nonlinearity association between vitamin D level and all-cause mortality. All data analyses were performed using R version 3.6.3, and *P* values less than 0.05 indicated statistical significance.

## 3. Results

### 3.1. Subject Characteristics

The baseline characteristics of all DKD participants grouped by vitamin D status are presented in Table 1. The overall mean (SD) 25(OH)D concentration was 55.9 (26.3). According to the Endocrine Society Clinical Practice Guidelines, 552 (45.9%) participants were in the vitamin D deficiency group (25(OH)D < 50 nmol/l), 409 (34.0%) participants were in the vitamin D insufficiency group (50 ≤ 25(OH)D < 75 nmol/l), and 241 participants (20.0%) were in the vitamin D sufficiency group (25(OH)D ≥ 75 nmol/l). The overall mean (SD) of participants with DKD aged 65.0 (12.6). Participants with DKD who had higher serum 25(OH)D levels were more commonly male and non-Hispanic white, were more likely to be older, and had lower BMI and higher education levels.

### 3.2. Association between Vitamin D Level and Mortality

**Figure 1:**
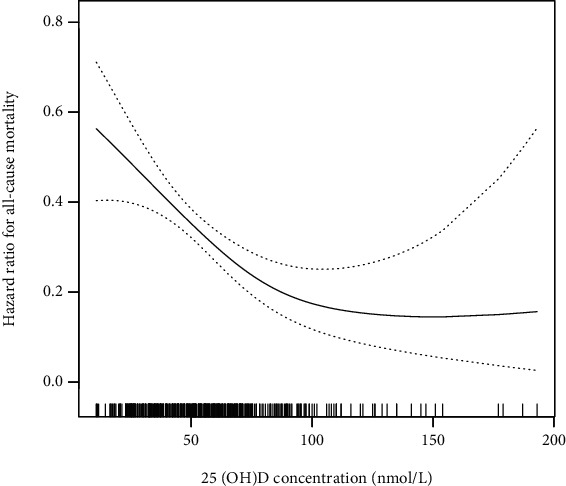
The association between vitamin D level and all-cause mortality. A nonlinear relationship between them was detected after adjusting for age, sex, race, BMI, education levels, family income-poverty ratio, glucose, glycohemoglobin, total cholesterol, HDL, LDL, triglyceride, creatinine, and blood urea nitrogen. The solid line and dashed line represent the estimated values and their corresponding 95% confidence intervals.

**Figure 2:**
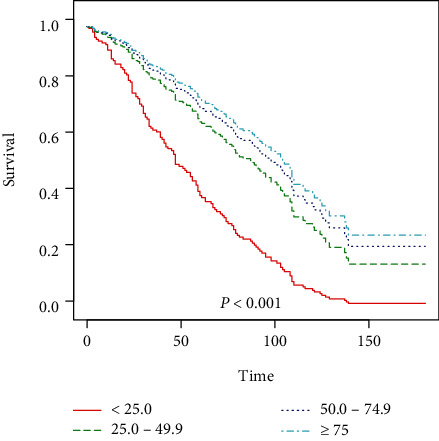
Kaplan–Meier survival analysis plot for all-cause mortality with serum 25(OH)D categories (<25.0, 25.0 to <50, 50.0 to <75, and ≥75 nmol/l). DKD patients in 25(OH)D < 25.0 nmol/l had the highest probability of survival, and patients in 25(OH)D ≥ 75 nmol/l had the lowest probability of survival.

Table 2 shows the HRs for all-cause mortality in DKD patients based on different quartiles of vitamin D level. After adjustment for age, sex, and race for model 1, the multivariate-adjusted HRs (95% CIs) from the lowest 25(OH)D group to the highest 25(OH)D group (<25.0, 25.0 to <50, 50.0 to <75, and ≥75 nmol/l) were 1.00 (reference), 0.60 (0.42, 0.86), 0.46 (0.32, 0.67), and 0.52 (0.34, 0.78) for all-cause mortality, respectively (*P* for trend = 0.003) (Table 2). For per one-unit increment in natural log-transformed 25(OH)D level, there was an 18% lower risk of all-cause mortality (Table 2). After further adjusted (from model 1) for BMI, education levels, and family income-poverty ratio for model 2, the multivariate-adjusted HRs (95% CIs) from the lowest 25(OH)D group to the highest 25(OH)D group (<25.0, 25.0 to <50, 50.0 to <75, and ≥75 nmol/l) were 1.00 (reference), 0.63 (0.42, 0.95), 0.51 (0.34, 0.78), and 0.55 (0.34, 0.88) for all-cause mortality, respectively (*P* for trend = 0.021) (Table 2). For per one-unit increment in natural log-transformed 25(OH)D level, there was a 15% lower risk of all-cause mortality (Table 2). Model 3 had similar results after additional adjustment from model 2 for glucose, glycohemoglobin, total cholesterol, HDL, LDL, triglyceride, creatinine, and blood urea nitrogen; the multivariate-adjusted HRs (95% CIs) from the lowest 25(OH)D group to the highest 25(OH)D group (<25.0, 25.0 to <50, 50.0 to <75, and ≥75 nmol/l) were 1.00 (reference), 0.45 (0.25, 0.83), 0.37 (0.20, 0.69), and 0.33 (0.16, 0.68) for all-cause mortality, respectively (*P* for trend = 0.010) (Table 2). Per one-unit increment in natural log-transformed 25(OH)D level was associated with a 26% reduced risk of all-cause mortality (Table 2). Moreover, a nonlinear (U-shaped) relationship between continuous variable of vitamin D level and all-cause mortality was detected a generalized additive model after adjusting for age, sex, race, BMI, education levels, family income-poverty ratio, glucose, glycohemoglobin, total cholesterol, HDL, LDL, triglyceride, creatinine, and blood urea nitrogen. Smooth curve fitting graph illustrated the risk of all-cause mortality significantly decreased with the increase concentration of 25(OH)D in DKD patients (*P* = 0.001) (Figure 1). Finally, we used Kaplan–Meier survival analysis for all-cause mortality with vitamin D categories (<25.0, 25.0 to <50, 50.0 to <75, and ≥75 nmol/l). The curves demonstrate that patients 25(OH)D < 25.0 nmol/l were associated with the lowest lifetime risk of all-cause mortality (Figure 2). We then analyzed the risk of death with or without accidents (unintentional injuries) and malignant neoplasm cause according to serum 25(OH)D concentrations among patients with DKD using a competing risk regression model (Table 3). After adjustments in the multivariable Cox and competing risk analyses, results of analyses observed in model 1, model 2, and model 3 for nonaccident- and malignant neoplasm-cause mortality were consistent with core results of the multivariable Cox and noncompeting risk analyses (Table 3).

## 4. Discussion

Numerous observational studies demonstrated that vitamin D insufficiency/deficiency was independently correlated with an elevated risk of developing DKD events in diabetic patients [19–22]. Some studies have shown that low levels of vitamin D and vitamin D resistance were classical features of chronic kidney disease [23], and some studies found that CKD is a risk factor for 25D deficiency [24]. Also, some studies found that vitamin D supplementation showed to be beneficial in preventing the progression of DKD. However, there was little evidence on vitamin D status and risk of mortality in DKD patients [25, 26]. Our analysis using NHANES 2001–2014 data, for the first time, found that elevated vitamin D levels were linearly and significantly correlated with lower risk of all-cause mortality among DKD patients. Our analysis was still robust after adjustment for potential confounders, indicating that serum vitamin D concentrations may be independently correlated with all-cause mortality in DKD patients.

Recent studies from NHANES observed higher vitamin D levels were significantly correlated with decreased risks of all-cause and CVD-related mortality among patients with diabetes and prediabetes [17, 27]. DKD, a major complication of diabetes, enhances the risk of mortality and is the common cause of end-stage renal disease. Therefore, further investigations may be essential for the link between vitamin D concentrations and survival status among individuals with DKD. Inflammation plays an important role in the progression of diabetes, which eventually leads to DKD [28]. Growing evidence found that low serum vitamin D levels were significantly correlated with adhesion factors and inflammatory mediators, including interleukin 6 (IL-6), c-reactive protein (CRP), VCAM-1, and ICAM-1. For example, Manion et al. reported that patients who suffered from vitamin D deficiency displayed 23% higher levels of IL-6 compared to those who had normal vitamin D levels [29]. Mao et al. reported that vitamin D deficiency or insufficiency had elevated inflammation markers in patients with DKD, such as TNF-*α*, IL-6, and ICAM-1 [30]. Eleftheriadis et al. suggested that vitamin D analogue decreased IL-6 and TNF-*α* expression levels in peripheral blood mononuclear cells (PBMCs) of healthy volunteer [31]. Lucisano et al. demonstrated that chronic kidney disease patients displayed lower vitamin D levels and higher expression of inflammatory markers, and vitamin D supplementation induced a significant reduction of IL-6, IL-17, IFN-*ϒ*, TNF-*α*, and IL-1*β* in these patients [32]. These findings indicate that vitamin D may diminish the secretion of inflammatory cytokines and inflammatory cell infiltration, thereby exerting a strong renal protective effect.

Proteinuria is an early biomarker and a major risk indicator of DKD progression. Thus, it is of great clinical significance to explore the renal protection and slow down DKD progression by reducing proteinuria. A cross-sectional study from NHANES observed a strong inverse correlation between vitamin D levels and proteinuria, indicating that vitamin D has effects on antiproteinuria [33]. Some researchers also described that vitamin D supplementation significantly reduced proteinuria in type II diabetes patients [34, 35]. Vitamin D supplementation also had similar effect in type I diabetes patients [36]. Podocyte injury is closely related to proteinuria. Kuhlmann et al. found that vitamin D reduces proteinuria and glomerulosclerosis by inhibiting podocyte injury in an animal study [37]. Trohatou et al. demonstrated that vitamin D supplementation contributed to attenuation of podocytic damage by influencing the nephrin-PI3K-Akt signaling pathway [38]. Other studies also found that some signaling pathways, such as Wnt signaling pathway and PI3K/Akt signaling pathway, are involved in the improving effect of vitamin D on podocyte injury [39, 40]. These findings indicate vitamin D favorable effects on proteinuria. Vitamin D supplementation significantly reduced inflammatory factors and proteinuria in patients, suggesting that vitamin D supplementation was of great significance in reducing the mortality of DKD.

Our study has strengths and limitations. To the best of the authors' knowledge, our study is the first to examine the link between vitamin D levels and risk of mortality in DKD patients. In addition, the strengths of this study include NHANES study with nationally representative samples with DKD and multiple potential confounding factors were adjusted to detect relative associations. Limitations should be also mentioned. First, our analysis was a cross-sectional study, with data collected at a point in time or over a very short period. However, Bushnaq et al. also used cross-sectional studies to look at some issues related to vitamin D [41]. Batacchi et al. also chose to measure vitamin D levels at a single time point when studying the effects of vitamin D2 supplementation on health and vitamin D3 metabolism in patients with chronic kidney disease [42]. Therefore, only the data at a single time point in our study are in line with the design principle, but there were indeed limitations in the data at a single time point. Second, vitamin D concentration was measured at a single time point in epidemiologic studies, which might not accurately reflect patients' long-term and seasonal vitamin D level. Third, though we have adjusted for many potential confounding factors, the potential effect of unmeasured confounders (including exposure to direct sunlight, sunscreen use, and vitamin D intake) could not be entirely ruled out. Fourth, we found no clinical data related to corticosteroids, orlistat, statins, and thiazide diuretics in the NHANES database; although these drugs are known to affect vitamin D levels, we were unable to consider (or adjust for) the effects of the drugs, which was a flaw in our study.

In total, evidence was found indicating DKD patients who had elevated vitamin D levels significantly correlated with lower all-cause mortality. This finding indicated that maintaining adequate vitamin D levels has potential advantages in the primary prevention of mortality among individuals with DKD.

## Figures and Tables

**Table 1:** Baseline characteristics of participants with DKD according to serum 25(OH)D concentrations in NHANES 2001–2014.

Variables	Serum 25(OH)D concentrations (nmol/l)				*P* value
	<25.0	25.0 to <50	50.0 to <75	≥75	
Number of patients	102	450	409	241	
Age (years)	60.5 ± 13.2	63.6 ± 12.7	65.7 ± 12.9	68.3 ± 10.7	<0.001
BMI (kg/m^2^)	34.0 ± 8.4	33.2 ± 8.5	31.4 ± 6.9	31.1 ± 6.8	<0.001
Sex (%)					0.043
Male	48 (47.1%)	247 (54.9%)	251 (61.4%)	135 (56.0%)	
Female	54 (52.9%)	203 (45.1%)	158 (38.6%)	106 (44.0%)	
Race (%)					<0.001
Non-Hispanic white	18 (17.6%)	108 (24.0%)	168 (41.1%)	117 (48.5%)	
Non-Hispanic black	58 (56.9%)	169 (37.6%)	87 (21.3%)	64 (26.6%)	
Mexican American	19 (18.6%)	121 (26.9%)	80 (19.6%)	24 (10.0%)	
Other	7 (6.9%)	52 (11.6%)	74 (18.1%)	36 (14.9%)	
Education levels					0.002
Less than high school	46 (45.1%)	219 (48.7%)	192 (46.9%)	78 (32.4%)	
High school or equivalent	21 (20.6%)	97 (21.6%)	90 (22.0%)	71 (29.5%)	
College or above	34 (33.3%)	131 (29.1%)	126 (30.8%)	91 (37.8%)	
Other	1 (1.0%)	3 (0.7%)	1 (0.2%)	1 (0.4%)	
Family income-poverty ratio					0.582
≤1.0	32 (34.4%)	119 (29.4%)	99 (25.8%)	56 (25.6%)	
1.0–3.0	43 (46.2%)	186 (45.9%)	193 (50.4%)	109 (49.8%)	
>3	18 (19.4%)	100 (24.7%)	91 (23.8%)	54 (24.7%)	

**Table 2:** HR (95% CIs) for all-cause mortality according to serum 25(OH)D concentrations among patients with DKD in NHANES 2001–2014.

Model	Serum 25(OH)D concentrations (nmol/l)				*P* trend	Per one-unit increment in natural log-transformed 25(OH)D
	<25.0	25.0–49.9	50.0–74.9	≥75		
Model 1^∗^	1.00	0.60 (0.42, 0.86)	0.46 (0.32, 0.67)	0.52 (0.34, 0.78)	0.003	0.82 (0.73, 0.93)
Model 2^†^	1.00	0.63 (0.42, 0.95)	0.51 (0.34, 0.78)	0.55 (0.34, 0.88)	0.021	0.85 (0.73, 0.97)
Model 3^‡^	1.00	0.45 (0.25, 0.83)	0.37 (0.20, 0.69)	0.33 (0.16, 0.68)	0.010	0.74 (0.59, 0.93)

^∗^Model 1: adjusted for age, sex, and race. ^†^Model 2: further adjusted (from model 1) for BMI, education levels, and family income-poverty ratio. ^‡^Model 3: further adjusted (from model 2) for glucose, glycohemoglobin, total cholesterol, HDL, LDL, triglyceride, creatinine, and blood urea nitrogen.

**Table 3:** Competing risk of death with or without accident (unintentional injuries) and malignant neoplasm cause according to serum 25(OH)D concentrations among patients with DKD in NHANES 2001–2014.

Model	Serum 25(OH)D concentrations (nmol/l)				*P* trend
	<25.0	25.0–49.9	50.0–74.9	≥75	
Model 1^∗^	1.0	0.59 (0.40, 0.85)	0.45 (0.31, 0.67)	0.45 (0.31, 0.67)	<0.01
Model 2^†^	1.0	0.60 (0.41, 0.87)	0.45 (0.30, 0.67)	0.49 (0.31, 0.76)	<0.01
Model 3^‡^	1.0	0.66 (0.45, 0.98)	0.47 (0.31, 0.71)	0.49 (0.31, 0.76)	<0.01

^∗^Model 1: adjusted for age, sex, and race. ^†^Model 2: further adjusted (from model 1) for BMI, education levels, and family income-poverty ratio. ^‡^Model 3: further adjusted (from model 2) for glucose, glycohemoglobin, total cholesterol, HDL, LDL, triglyceride, creatinine, and blood urea nitrogen.

## Data Availability

All data generated or analyzed during this study are available from the corresponding author on reasonable request.
